# Ten Years of Congenital Zika Syndrome: From Outbreak to a Decade of Clinical, Therapeutic, and Preventive Advances in a Tropical Disease Context

**DOI:** 10.3390/tropicalmed11050124

**Published:** 2026-05-06

**Authors:** Fabrício Silva Pessoa

**Affiliations:** Pediatric Infectious Diseases Department, UFMA University Hospital, Barão de Itapary, 227, Centro, São Luís 65080-805, MA, Brazil; fabriciosilvapessoa@hotmail.com; Tel.: +55-98-9-8158-3800

**Keywords:** Zika virus, congenital Zika syndrome, microcephaly, flavivirus, tropical disease, neurodevelopment, ZIKV vaccine, *Aedes aegypti*, arbovirus, neglected tropical diseases

## Abstract

A decade has elapsed since the first recognized cluster of congenital anomalies associated with Zika virus (ZIKV) was reported in Brazil in 2015, culminating in the formal delineation of Congenital Zika Syndrome (CZS) as a specific pattern of birth defects. This narrative review examines the ten-year trajectory of CZS as a tropical infectious disease, from its initial emergence and public health emergency declaration by the World Health Organization (WHO) in February 2016, through evolving epidemiological, clinical, and scientific understanding. CZS is characterized by a spectrum of severe neurological manifestations—including microcephaly, subcortical calcifications, malformations of cortical development, ventriculomegaly, and corpus callosum abnormalities—alongside ophthalmic, auditory, and musculoskeletal complications. Transmitted primarily by *Aedes aegypti* mosquitoes in tropical and subtropical regions, ZIKV disproportionately affects low- and middle-income countries in Latin America, Africa, and Southeast Asia, underscoring its nature as a quintessential tropical disease linked to poverty, inadequate vector control, and health inequity. Over ten years, substantial advances have been made in understanding ZIKV pathogenesis, neurodevelopmental outcomes, diagnostic criteria, and multidisciplinary clinical management of affected children. In the therapeutic and preventive domain, over 45 vaccine candidates have been identified, with 16 reaching Phase 1 or 2 clinical trials by late 2025, though no licensed vaccine or specific antiviral therapy yet exists. This review contextualizes CZS within the broader framework of neglected tropical diseases, evaluates its global and family-level burden, and critically appraises progress and remaining gaps in clinical care, vaccination, and vector control over the past ten years.

## 1. Introduction

The emergence of Zika virus (ZIKV) as a major teratogen represents one of the most significant and unexpected events in modern tropical medicine. Although ZIKV was first isolated in 1947 from a rhesus macaque in the Zika Forest of Uganda and subsequently identified in humans in Nigeria in 1952, it remained a pathogen of modest clinical concern for decades, associated with mild, self-limited febrile illness [1,2].

The first recognized human outbreak occurred in the Yap Islands of Micronesia in 2007, affecting an estimated 73% of the island population, followed by a larger epidemic in French Polynesia in 2013–2014, during which Guillain–Barré syndrome (GBS) cases were first associated with ZIKV infection [3,4].

In May 2015, Brazilian health authorities reported the first confirmed autochthonous ZIKV cases in the northeast region. By late 2015, an unprecedented surge in microcephaly cases—predominantly in the northeastern states of Brazil—prompted an intensive national and international scientific response. On 28 November 2015, Brazil declared a national public health emergency. On 1 February 2016, the World Health Organization (WHO) declared ZIKV infection a Public Health Emergency of International Concern (PHEIC) [5,6].

The constellation of congenital anomalies associated with prenatal ZIKV exposure was formally characterized in 2017 as Congenital Zika Syndrome (CZS), defined by a specific pattern including severe microcephaly with a partially collapsed skull, thin cerebral cortex with subcortical calcifications, macular scarring and focal pigmentary retinal mottling, congenital contractures of varying severity, and early-onset hypertonia with extrapyramidal symptoms. These features were recognized as distinguishing CZS from other congenital infections and defining it as a novel teratogenic syndrome [7].

Ten years after the first CZS cases, this narrative review synthesizes the epidemiological, clinical, neurodevelopmental, diagnostic, therapeutic, and preventive advances achieved during this period, contextualizing CZS within the framework of tropical infectious diseases and examining the social determinants that render tropical populations disproportionately vulnerable to ZIKV and its congenital consequences.

## 2. Background: Zika Virus as a Tropical Pathogen

### 2.1. Virology and Transmission

Zika virus is a positive-sense, single-stranded RNA virus of the genus *Flavivirus*, family *Flaviviridae*, phylogenetically related to dengue (DENV), West Nile, yellow fever, and Japanese encephalitis viruses. The ZIKV genome encodes three structural proteins—capsid (C), precursor membrane (prM), and envelope (E)—and seven nonstructural proteins (NS1, NS2A, NS2B, NS3, NS4A, NS4B, NS5). Two major lineages are recognized: the African lineage and the Asian lineage; the latter was responsible for the Pacific and American epidemics associated with congenital disease [8,9].

The predominant transmission route is through the bite of infected *Aedes* mosquitoes, principally *Aedes aegypti* and, to a lesser extent, *Aedes albopictus*. Both species thrive in tropical and subtropical environments characterized by warm temperatures, high humidity, and standing water—conditions frequently associated with rapid urbanization, inadequate infrastructure, and limited waste management, firmly placing ZIKV among the quintessential arboviruses of tropical medicine [10,11].

Beyond vector-borne transmission, ZIKV is unique among flaviviruses for documented non-vectorial routes including sexual transmission (male-to-female and female-to-male), vertical (mother-to-fetus) transmission—the primary mechanism underlying CZS—and probable transmission through blood transfusion and, in limited reports, breast milk. Sexual transmission and prolonged viral persistence in seminal fluid, documented for up to 69 days after symptom onset, have added complexity to preventive strategies [12,13].

### 2.2. Epidemiology and Global Burden

From 2015 to 2016, ZIKV spread rapidly across the Americas, with local transmission documented in over 48 countries and territories. Brazil bore the heaviest burden; between November 2015 and October 2018, approximately 3420 CZS cases were confirmed by the Brazilian Ministry of Health. Globally, approximately 4000 children across 27 countries have been affected by CZS [5,14].

Epidemiological surveillance has established that the trimester of maternal infection is the principal determinant of fetal risk. First-trimester exposure carries the highest risk for severe fetal neurological compromise, with reported incidence of microcephaly ranging from 1% to 13% following confirmed maternal infection in the first trimester, compared with substantially lower risks in the second and third trimesters [15,16].

Since 2017, ZIKV incidence has markedly declined in the Americas, attributed to the combination of herd immunity in heavily affected populations and seasonal fluctuations in vector activity. Nonetheless, serological surveillance demonstrates continued low-level circulation in Brazil, Colombia, and parts of the Caribbean. Localized outbreaks have continued in India, Angola, and Southeast Asian nations. The combination of large susceptible populations in tropical regions and expanding vector habitat driven by climate change maintains the threat of future large-scale outbreaks [17,18].

ZIKV disproportionately affects populations living in poverty, in areas with high vector density, limited healthcare access, and inadequate reproductive health services—an epidemiological profile mirroring that of neglected tropical diseases (NTDs), with which ZIKV shares vector dependence, geographic concentration in tropical low- and middle-income countries, and the compounding effects of poverty and health inequity [19].

## 3. Clinical Profile of Congenital Zika Syndrome

### 3.1. Neurological Manifestations

The neurological phenotype of CZS is its defining and most severe dimension. Neuroimaging studies (computed tomography and MRI) have revealed a consistent pattern: (i) microcephaly, defined as head circumference more than 2 standard deviations (SD) below the mean for gestational age and sex, with a distinctive partially collapsed skull; (ii) parenchymal and subcortical calcifications, particularly at the gray–white matter junction and in the basal ganglia; (iii) malformations of cortical development, including lissencephaly, pachygyria, and simplified gyral pattern; (iv) ventriculomegaly; and (v) brainstem and cerebellar hypoplasia [7,20,21].

The molecular basis of these findings involves direct ZIKV infection of neural progenitor cells (NPCs) in the hippocampus and developing cortex, leading to cell cycle disruption, apoptosis, and impaired neurogenesis. Positron emission tomography studies in animal models have demonstrated global neuroinflammation in ZIKV infection. Yuan et al. demonstrated that a single mutation in the prM protein of the Asian lineage ZIKV enhances NPC tropism, contributing to the greater teratogenicity observed in the American epidemic strain compared with earlier African strains [22,23].

Epilepsy is a major complication, affecting 48–83% of microcephalic infants in some series, often presenting as infantile spasms or multifocal epilepsy refractory to standard anticonvulsant therapy. Van der Linden et al. (2018) documented the epilepsy profile in a cohort of 57 infants with CZS, finding that 46% had seizures within the first year of life, with 89% of those seizure cases resistant to at least two anticonvulsant medications [24].

### 3.2. The Spectrum of CZS: From Severe Disease to Asymptomatic Exposure

A critically important clinical observation from longitudinal Brazilian cohorts is that CZS exists on a spectrum of severity that must be carefully distinguished. At one end are infants with confirmed microcephaly and brain abnormalities at birth, who have a profoundly severe prognosis—virtually all develop cerebral palsy, and most show near-floor performance across all developmental domains. At the other end are infants exposed to ZIKV in utero who are born without microcephaly and without brain or eye abnormalities on imaging; in these children, neurodevelopmental trajectories are largely within the normal range [25,26].

Between these poles lies a third, clinically critical group: infants who appear neurologically normal at birth, but who later develop postnatal-onset microcephaly, seizures, or neuromotor delay within the first months of life. França et al. (2016), in the first 1501 confirmed CZS livebirths in Brazil, documented that a significant proportion of infants without microcephaly at birth subsequently developed head circumference decline postnatally. This phenomenon—the ‘hidden burden’—arises because the destructive neurological process may not manifest as microcephaly until after birth, when brain growth fails to keep pace with normal skull expansion. It does not imply that all asymptomatic-at-birth infants will deteriorate; rather, it identifies a subgroup requiring close surveillance [5,27].

This distinction is clinically fundamental: the high prevalence of apparently normal appearance at birth (estimated at 65–83% of exposed infants) can delay CZS diagnosis if surveillance relies solely on microcephaly identification at birth, and should not be conflated with the favorable prognosis seen in those exposed without any brain abnormalities [26].

### 3.3. Ophthalmological, Auditory, and Musculoskeletal Manifestations

Ophthalmic involvement in CZS is well-established, with macular scarring and focal pigmentary retinal mottling recognized as cardinal features. Ventura et al. (2016) described ophthalmic findings in 29 of 70 infants (41.4%) with microcephaly and confirmed or suspected ZIKV exposure, including chorioretinal atrophy and optic disc hypoplasia. Microphthalmia and cataracts are less common but documented findings. Ophthalmic evaluation is therefore mandatory for all infants with confirmed or suspected ZIKV exposure [28].

Sensorineural hearing loss has been documented in approximately 6% of CZS-affected infants in prospective cohort studies. Leal et al. (2016) reported hearing loss in 6 of 70 (8.6%) microcephalic infants with ZIKV evidence using auditory brainstem response (ABR) testing. Musculoskeletal manifestations, including arthrogryposis and hypertonia-related limb deformities, are observed particularly in severely affected infants and significantly complicate rehabilitation efforts [29,30].

### 3.4. Neurodevelopmental Outcomes over Ten Years of Follow-Up

Longitudinal follow-up data from Brazilian cohorts, published between 2017 and 2024, have comprehensively characterized neurodevelopmental trajectories. In the Cohort of Infants with Microcephaly (CIPESPE) from Recife, Pernambuco, children with severe CZS (microcephaly with brain abnormalities) demonstrated performance at or near the floor of all developmental assessments—including the Bayley Scales of Infant and Toddler Development—with profound deficits in gross motor, fine motor, cognitive, language, and social-emotional domains at 18 and 36 months of age [25,31].

Vianna et al. (2020), in an 18-month follow-up of 59 CZS-exposed children, documented that those without brain or eye abnormalities had largely normal developmental outcomes, reinforcing the prognostic importance of neuroimaging findings at birth. Head circumference trajectories in these cohorts confirm that Z-scores for head circumference often decrease in the first 6 months of life, even in infants born without microcephaly, highlighting the need for serial measurements throughout infancy [32].

## 4. Diagnostic Advances

### 4.1. Laboratory Diagnosis

Laboratory confirmation of ZIKV infection relies on molecular and serological methods. Reverse transcription polymerase chain reaction (RT-PCR) of serum, urine, amniotic fluid, or cerebrospinal fluid constitutes the gold standard for confirming acute or recent ZIKV infection. Viremia is transient (detectable for approximately 5–7 days after symptom onset), but urine RT-PCR extends the diagnostic window by several days. Deckard et al. (2016) demonstrated that urine specimens are more consistently positive than serum samples in the acute phase [33].

Cross-reactivity between ZIKV and other flaviviruses—particularly DENV and Yellow Fever virus—significantly limits serological assay specificity. IgM ELISA (ZIKV MAC-ELISA) has sensitivity of 80–90% but specificity of only 65–75% in flavivirus-endemic areas. Plaque reduction neutralization testing (PRNT) offers improved specificity but is technically demanding and not universally available. Over the past decade, multiplex molecular platforms capable of simultaneously detecting ZIKV, DENV, and chikungunya virus have been developed and validated, substantially improving differential diagnosis in co-endemic regions [34,35].

### 4.2. Prenatal and Neonatal Evaluation

Fetal ultrasound surveillance is the cornerstone of prenatal monitoring in ZIKV-exposed pregnancies. Ultrasound findings associated with CZS include microcephaly, intracranial calcifications, ventriculomegaly, and decreased brain volume. Fetal MRI provides superior cortical characterization and has been used in specialized centers to identify malformations of cortical development not visualized on ultrasound. Cauchemez et al. (2016) estimated the risk of microcephaly following ZIKV infection in the first trimester at approximately 1%, while subsequent Brazilian data suggested higher risks of 4–13% in the first trimester [15,36].

For neonates, comprehensive evaluation protocols recommended by the Brazilian Ministry of Health, the U.S. CDC, and WHO include: head circumference measurement, complete neurological examination, cranial CT or MRI, ophthalmological examination, auditory brainstem response testing, and RT-PCR/serological confirmation. These standardized protocols have enabled systematic CZS identification and longitudinal follow-up data generation at reference centers [37].

## 5. Clinical Management and Multidisciplinary Care

In the absence of a specific antiviral therapy, CZS management is fundamentally supportive and multidisciplinary. Coordinated care involves neonatology, pediatric neurology, rehabilitation medicine, ophthalmology, audiology, physiotherapy, speech-language pathology, occupational therapy, nutritional support, and social work. Early intervention programs, initiated within the first months of life, aim to optimize developmental outcomes through intensive habilitation [38].

Anticonvulsant therapy is central to management for the substantial proportion with refractory epilepsy. Vigabatrin and ACTH have been used for infantile spasms, while polytherapy is frequently required. The ketogenic diet has been explored as adjunctive therapy given frequent pharmacoresistance. Management of spasticity and dystonia includes physiotherapy, botulinum toxin injections, oral baclofen, and intrathecal baclofen pump implantation in selected patients [24,38].

Nutritional management is critical, as dysphagia and gastroesophageal reflux are common. Gastrostomy tube placement is frequently necessary in severely affected children to ensure adequate caloric intake and prevent aspiration pneumonia. Orthopedic interventions—including serial casting, orthoses, and surgical correction of contractures—are required in a significant number of patients with arthrogryposis [38].

The psychosocial burden on families—predominantly mothers—has been extensively documented. Studies using validated psychiatric instruments have documented high rates of depression (50–70%), anxiety, post-traumatic stress disorder, and social isolation among caregivers of CZS-affected children in Brazil. Integrative family support programs including psychosocial counseling and social protection measures are recognized as essential components of comprehensive CZS care [39].

## 6. Therapeutic and Preventive Advances

### 6.1. Antiviral Drug Development

No specific antiviral agent for ZIKV has received regulatory approval as of early 2026. Drug repurposing screens identified several candidate compounds with in vitro anti-ZIKV activity, including sofosbuvir (NS5B polymerase inhibitor), ribavirin, favipiravir, and antiparasitic agents such as ivermectin and chloroquine. However, none has demonstrated clinical efficacy in human trials, and concerns regarding teratogenicity preclude the use of several candidates in pregnant women [40].

Host-directed therapeutic approaches have targeted cellular pathways exploited by ZIKV, including the AXL receptor tyrosine kinase—a major ZIKV entry receptor on NPCs—the autophagy pathway, and the interferon signaling cascade. Wang et al. (2017) developed a bispecific human monoclonal antibody (mAb) against ZIKV envelope protein domain III that demonstrated potent in vitro neutralization and protection in murine models. Passive transfer of anti-envelope antibody fractions has protected mice from lethal ZIKV challenge in multiple studies, establishing mAbs as a promising avenue for both therapeutic and pre-exposure prophylactic application [41,42].

### 6.2. Vaccine Development

Following the 2016 emergency, over 45 vaccine candidates were identified within a two-year period, spanning DNA, mRNA, inactivated whole-virus, live-attenuated, viral vector-based, virus-like particle (VLP), and recombinant subunit platforms. The immunodominant antigen across platforms is the envelope (E) protein, with the prM-E combination used in most leading candidates [43,44].

DNA vaccine candidates developed at NIAID (VRC5283 and VRC5288) expressed prM-E sequences and elicited robust neutralizing antibody responses in Phase 1 trials in flavivirus-naïve and seropositive participants, with a two-dose schedule maintaining neutralizing antibody titers for at least 12 months after the last dose. Dowd et al. (2016) published the initial Phase 1 safety and immunogenicity results for VRC5283 in the New England Journal of Medicine [45].

mRNA-based vaccines have advanced rapidly following COVID-19 platform validation. Moderna’s mRNA-1893—encoding prM-E using a nucleoside-modified mRNA encapsulated in lipid nanoparticles—completed Phase 2 evaluation in approximately 800 flavivirus-seropositive and seronegative adults in the United States and Puerto Rico (NCT04917861). Richner et al. (2017) first demonstrated that nucleoside-modified mRNA-LNP vaccines expressing prM-E conferred complete protection from lethal ZIKV challenge in immunocompetent mice and rhesus macaques, and subsequently protected against ZIKV-induced congenital disease in murine pregnancy models [46,47].

Inactivated vaccine candidates include ZPIV (purified formalin-inactivated ZIKV, developed by WRAIR and Sanofi Pasteur) and VLA1601 (adjuvanted, aluminum hydroxide-adsorbed purified inactivated candidate, Valneva)—currently the most clinically advanced Zika vaccine, with an additional Phase 1 trial (VLA1601-102) initiated in March 2024 with initial results published in November 2025. As of late 2025, 16 vaccine candidates were in Phase 1 or 2 clinical trials and three mAb candidates were in Phase 1 trials. Approximately USD 350 million in cumulative research funding has been mobilized for ZIKV vaccine development since 2016 [43,48].

Despite this progress, no vaccine has advanced to Phase 3 efficacy trials. The dramatic decline in ZIKV incidence since 2017 prevents powering Phase 3 trials for prevention of infection or CZS under low natural transmission conditions. Regulatory pathways under discussion include the FDA Animal Rule, human challenge studies, and surrogate endpoint approaches. Additional challenges include: (i) the potential for antibody-dependent enhancement (ADE) given cross-reactive immune responses—most extensively studied with DENV, but also documented in the context of West Nile virus and Japanese encephalitis virus antibodies cross-reacting with ZIKV epitopes on the envelope domain II fusion loop—a region of high structural conservation across multiple flaviviruses, raising concerns that pre-existing immunity to any co-circulating flavivirus could theoretically modulate ZIKV infection outcomes; (ii) ethical complexities of including pregnant women and children in vaccine trials; and (iii) diminished commercial incentive in the absence of sustained outbreaks [49,50].

### 6.3. Vector Control and Preventive Strategies

In the absence of a licensed vaccine, prevention relies on vector control and behavioral risk reduction. Conventional vector control—source reduction, larviciding, and adult mosquito control with insecticides—is limited by operational challenges in urban settings and insecticide resistance in *Aedes aegypti* populations, documented in Brazil, Colombia, and several Asian countries [51].

Biological control strategies have gained prominence, most notably *Wolbachia*-infected *Aedes aegypti* deployment. The wMel strain of *Wolbachia* reduces ZIKV replication within mosquitoes and decreases vector competence. A cluster-randomized trial in Yogyakarta, Indonesia demonstrated a 77.1% reduction in dengue incidence in *Wolbachia*-release areas (Utarini et al., 2021). Their impact on ZIKV transmission, while biologically plausible, has not been directly measured in efficacy trials [52].

Release of genetically modified *Aedes aegypti* (Oxitec OX513A, carrying a self-limiting gene) has demonstrated significant local population suppression in Brazilian and Cayman Islands field trials. Gene-drive approaches remain under evaluation for safety, ecological impact, and regulatory approval [53].

Behavioral risk reduction—including DEET/picaridin-based repellents, protective clothing, screened housing, and sexual transmission prevention—remains central to guidance for travelers and pregnant women in endemic regions. Preconception and antenatal counseling on ZIKV risk has been integrated into reproductive health services [37].

## 7. Congenital Zika Syndrome in the Context of Tropical Medicine

*Aedes aegypti* mosquitoes—ZIKV’s primary vector—place approximately 3.9 billion people in over 128 countries at risk for dengue alone; the same populations are equally exposed to ZIKV [54].

Climate change is projected to expand the geographic range of *Aedes* vector populations into previously non-endemic temperate regions. Mordecai et al. (2017) modeled that rising temperatures significantly extend the thermal window for ZIKV transmission, potentially expanding endemic transmission into parts of Southern Europe, the southeastern United States, and highland areas of Africa and South America [55].

The burden of CZS falls most heavily on women. Studies using validated instruments have documented that primary caregivers of CZS-affected children—predominantly mothers—experience high rates of clinical depression, anxiety, social isolation, and financial impoverishment due to lifelong caregiving demands and reduced labor force participation. This gender-differentiated burden has prompted calls for integrating CZS response into reproductive rights, women’s health, and disability rights frameworks [39].

## 8. Discussion

Ten years after the recognition of CZS, this review demonstrates a trajectory of remarkable scientific progress alongside sobering acknowledgment of persistent gaps in prevention, treatment, and equitable care delivery. The past decade has established CZS as a paradigmatic tropical teratogenic disease; elucidated ZIKV’s NPC tropism and neuroinflammatory mechanisms; characterized the full spectrum from severe microcephaly to largely normal neurodevelopment in non-microcephalic exposed infants; and advanced vaccine development to Phase 2 clinical trials across multiple platforms.

A key methodological consideration for this review is that much of the available evidence—particularly on early natural history, clinical spectrum, and family impact—derives from case series, cohort studies from a limited number of Brazilian reference centers, and review literature, given the novelty of CZS at the time of the epidemic. Over the past decade, randomized or controlled study designs have been applied primarily in vaccine and vector control research; clinical management evidence remains largely observational. This evidence base reflects the realities of studying a newly emerged teratogenic syndrome and underscores the need for continued prospective cohort studies as affected children enter adolescence.

The absence of a licensed vaccine or specific antiviral remains the most critical unresolved gap. The paradox of scientific success without clinical availability—driven by the epidemiological waning of ZIKV that undermines Phase 3 trial feasibility—demands regulatory innovation, including increased use of the FDA Animal Rule, surrogate endpoint frameworks, and international harmonization of Zika vaccine development pathways.

The longitudinal follow-up of severely affected children reinforces the imperative for sustained multidisciplinary care systems extending across childhood and into adulthood—systems that remain inadequately resourced in the low-income Brazilian northeastern states and other tropical regions where the burden is greatest. Investment in broad-spectrum antiviral strategies, platform vaccine technologies, integrated vector management, and health systems strengthening constitutes a rational response to tropical infectious disease preparedness in the current decade.

## 9. Conclusions

A decade of CZS research has transformed understanding of ZIKV teratogenesis and established a comprehensive clinical, diagnostic, and management framework for CZS-affected children. Key achievements include: characterization of the full spectrum of CZS from severe microcephaly to asymptomatic exposure; elucidation of ZIKV NPC tropism as the basis for teratogenesis; documentation of long-term neurodevelopmental outcomes across the severity spectrum; unprecedented vaccine pipeline development (16 Phase 1/2 candidates by 2025); and improved diagnostics for flavivirus-endemic settings.

Critical priorities for the next decade include: (1) licensure of a safe and effective ZIKV vaccine through innovative regulatory pathways; (2) development of antivirals suitable for use in pregnancy; (3) strengthened integrated vector control programs incorporating biological and genetic approaches; (4) expansion of equitable multidisciplinary care access in resource-limited settings; (5) sustained psychosocial and social protection support for affected families; and (6) robust surveillance systems capable of early detection of future ZIKV outbreaks. CZS remains a defining example of the intersection of tropical infectious disease, poverty, health inequity, and congenital disability—demanding sustained global scientific and political commitment.

## Data Availability

Data is contained within the article.
